# A bivariate genomic model with additive, dominance and inbreeding depression effects for sire line and three-way crossbred pigs

**DOI:** 10.1186/s12711-019-0486-2

**Published:** 2019-08-19

**Authors:** Ole F. Christensen, Bjarne Nielsen, Guosheng Su, Tao Xiang, Per Madsen, Tage Ostersen, Ingela Velander, Anders B. Strathe

**Affiliations:** 1 Department of Molecular Biology and Genetics, Center for Quantitative Genetics and Genomics, Aarhus University, Blichers Alle 20, 8830 Tjele, Denmark; 2 SEGES Pig Research Centre, Axeltorv 3, 1609 Copenhagen V, Denmark; 3 College of Animal Sciences and Technology, Huazhong Agricultural University, No. 1 Shizihan St., Hongshan District, Wuhan, 430070 Hubei People’s Republic of China; 4 Quantitative Clinical Pharmacology, Novo Nordisk A/S, Vandtårnsvej 108, 2860 Søborg, Denmark

## Abstract

**Background:**

Crossbreeding is widely used in pig production because of the benefits of heterosis effects and breed complementarity. Commonly, sire lines are bred for traits such as feed efficiency, growth and meat content, whereas maternal lines are also bred for reproduction and longevity traits, and the resulting three-way crossbred pigs are used for production of meat. The most important genetic basis for heterosis is dominance effects, e.g. removal of inbreeding depression. The aims of this study were to (1) present a modification of a previously developed model with additive, dominance and inbreeding depression genetic effects for analysis of data from a purebred sire line and three-way crossbred pigs; (2) based on this model, present equations for additive genetic variances, additive genetic covariance, and estimated breeding values (EBV) with associated accuracies for purebred and crossbred performances; (3) use the model to analyse four production traits, i.e. ultra-sound recorded backfat thickness (BF), conformation score (CONF), average daily gain (ADG), and feed conversion ratio (FCR), recorded on Danbred Duroc and Danbred Duroc-Landrace–Yorkshire crossbred pigs reared in the same environment; and (4) obtain estimates of genetic parameters, additive genetic correlations between purebred and crossbred performances, and EBV with associated accuracies for purebred and crossbred performances for this data set.

**Results:**

Additive genetic correlations (with associated standard errors) between purebred and crossbred performances were equal to 0.96 (0.07), 0.83 (0.16), 0.75 (0.17), and 0.87 (0.18) for BF, CONF, ADG, and FCR, respectively. For BF, ADG, and FCR, the additive genetic variance was smaller for purebred performance than for crossbred performance, but for CONF the reverse was observed. EBV on Duroc boars were more accurate for purebred performance than for crossbred performance for BF, CONF and FCR, but not for ADG.

**Conclusions:**

Methodological developments led to equations for genetic (co)variances and EBV with associated accuracies for purebred and crossbred performances in a three-way crossbreeding system. As illustrated by the data analysis, these equations may be useful for implementation of genomic selection in this system.

**Electronic supplementary material:**

The online version of this article (10.1186/s12711-019-0486-2) contains supplementary material, which is available to authorized users.

## Background

Crossbreeding is widely used in pig production because of the benefits of heterosis effects and breed complementarity. Commonly, sire lines, for example Duroc or Pietrain lines, are bred for production traits such as feed efficiency, growth and meat content, whereas maternal lines, for example Landrace and Yorkshire lines, are also bred for reproduction and longevity traits. The production traits give value to farmers in the finishing units, whereas the reproduction and longevity traits give value to farmers in the sow units. To benefit from heterosis effects in all production units, a crossbreeding system that is often used is a terminal three line system where two maternal lines are mated to produce crossbred sows, which then are mated to a sire line to produce three-way crossbred pigs for the production of meat. For example, in the DanBred production system, the maternal lines Danbred Landrace (L) and Danbred Yorkshire (Y) are mated to produce two-way crossbred Landrace–Yorkshire (LY) sows, which are then mated to sire line Danbred Duroc (D) boars, resulting in three-way crossbred Duroc-Landrace–Yorkshire (DLY) pigs.

Heterosis is caused by non-additive gene actions [1] and is one reason for the difference between performances of purebreds and crossbreds. In addition, there may be systematic differences in the environments of, for example, nucleus herds and finisher herds. For these reasons, selecting animals for high purebred performance may not result in selecting those that lead to high-performance crossbred offspring in a specific crossbreeding scheme. An approach to handle this is to define two breeding values for purebred animals, the usual breeding value for purebred performance (mating within breed) and an alternative breeding value for crossbred performance (mating with the other breed), as in Wei and van der Werf [2], and then select based on an index of the two estimated breeding values (EBV) [3, 4]. These two breeding values are correlated, with the correlation depending on the size of non-additive genetic effects in combination with differences in allele frequencies between lines, and possibly in addition, on genotype by environment interactions.

Non-additive genetic effects are usually not incorporated into models for genetic evaluation for at least two reasons. First, to accurately estimate dominance genetic variance and dominance genetic effects based on pedigree-based relationships, very large data sets with a high proportion of full-sibs are required [5]. Second, with pedigree-based relationships, specification and computation of non-additive genetic relationship matrices are difficult [6]. However, with genomic relationships instead of pedigree relationships, the story is different. Recently, pig breeding organisations have introduced genomic selection in their breeding schemes, and genetic evaluation is based on models that include genomic information. The focus has primarily been on additive genetic models and records on purebreds [7], but there has been increasing scientific interest in models with dominance genetic effects, and in combining purebred and crossbred information [8–10]. Genomic data provide more information for accurate estimation of dominance genetic effects and, in addition, including dominance effects into the models has become less difficult in practice, since computing the genomic dominance relationship matrix only requires knowledge about whether marker genotypes are heterozygous or not [8, 11].

For genomic evaluation in a crossbreeding system with two purebred lines and a crossbred F1 population, Vitezica et al. [12] introduced a flexible model, which contains both additive and dominance genotypic effects that are allowed to differ in purebreds and crossbreds, with correlations between them. Xiang et al. [13] extended the model to include genomic inbreeding depression effects that are viewed as resulting from directional dominance effects, and also presented equations for the genetic variances, genetic covariance and genetic correlation between purebred and crossbred performances as functions of the parameters in the model. Both papers used the model to analyse litter size in pigs, and provided estimates of correlations between genotypic effects in purebreds and crossbreds for both additive and dominance genotypic effects. In Xiang et al. [13], predictive performance for total genotypic effects was also investigated and it was found that including both inbreeding depression effects and dominance effects improved predictive performance. However, estimation of breeding values was not considered in these two papers. Moreover, to date, such a model has only been used on maternal lines and reproduction traits, and the model would need to be modified for production traits from a three-way crossing terminal system.

The aims of this study were to (1) present a modification of the model of Vitezica et al. [12] and Xiang et al. [13] for the case of a purebred sire line and three-way crossbred pigs; (2) based on this model, present equations for genetic variances, genetic covariance, and EBV for purebred and crossbred performance; (3) use the model to analyse four production traits, i.e. ultra-sound recorded backfat thickness, conformation score, average daily gain, and feed conversion ratio, recorded on Danbred Duroc and Danbred DLY pigs raised in the same environment; and (4) obtain estimates of genetic parameters, genetic correlations between purebred and crossbred performances, and EBV with associated accuracies for purebred and crossbred performances for this data set.

## Methods

In this section we present successively (1) the model for genomic evaluation in the terminal crossbreeding system with purebred sire line and three-way crossbred animals; (2) an equivalent single nucleotide polymorphism (SNP) effects model and equations for genetic variances and covariance; (3) equations for EBV with associated accuracies; (4) the data used in this study; and (5) details of the data analysis.

### Combined purebred and crossbred model

The bivariate model with phenotypes on the sire line and three-way crossbred pigs considered as two correlated traits is:

1 $$\begin{aligned} \mathbf {y}_{p}&=\mathbf {X}_{p}{\varvec{\beta }}_{p}+\mathbf {f}_p\eta _p+\mathbf {S}_p\mathbf {l}_p+\mathbf {u}_{p}+\mathbf {v}_{p}+\mathbf {e}_{p}, \nonumber \\ \mathbf {y}_{c}&= \mathbf {X}_{c}{\varvec{\beta }}_{c} +\mathbf {f}_c\eta _c+ \mathbf {S}_c\mathbf {l}_c+\mathbf {u}_{c} + \mathbf {v}_{c} + \mathbf {e}_{c}, \end{aligned}$$

where the first equation, with subscripts *p* for purebred, is the sire line part of the model, and the second equation, with subscripts *c* for crossbred, is the three-way crossbred part of the model. For the sire line part of the model, vector $\mathbf {y}_{p}$ contains phenotypes, vector ${\varvec{\beta }}_{p}$ contains fixed effects, matrix $\mathbf {X}_{p}$ relates animals with phenotypes to fixed effects, $\eta _{p}$ is the inbreeding depression per unit of genomic inbreeding, vector $\mathbf {f}_{p}$ is the vector of genomic inbreeding coefficients, vector $\mathbf {l}_p$ contains random litter effects, $\mathbf {l}_{p}\sim N(\mathbf {0},\sigma _{l,p}^{2}I)$ with $\mathbf {I}$ being the identity matrix, matrix $\mathbf {S}_p$ relates animals to litters, vector $\mathbf {u}_{p}$ contains additive genotypic effects that are assumed random, $\mathbf {u}_{p}\sim N(\mathbf {0},\sigma _{u,p}^{2}\mathbf {G}_{p})$ with $\mathbf {G}_{p}$ defined below, vector $\mathbf {v}_{p}$ contains dominance genotypic effects that are also assumed random, $\mathbf {v}_{p}\sim N(\mathbf {0},\sigma _{v,p}^{2}\mathbf {D}_{p})$ with $\mathbf {D}_{p}$ defined below, and $\mathbf {e}_{p}$ is the random residual error vector, $\mathbf {e}_{p}\sim N(\mathbf {0},\sigma _{e,p}^{2}\mathbf {I})$. For the three-way crossbred part of the model, notation and effects are defined as for the sire line part of the model.

The model is a modification of the model in Vitezica et al. [12] and Xiang et al. [13], where genetic effects $\mathbf {u}_{p}$, $\mathbf {u}_{c}$, $\mathbf {v}_{p}$ and $\mathbf {v}_{c}$ are “biological effects”. Additive and dominance relationships are specified across the sire line and the three-way crossbred pigs. The additive genomic relationship matrix is:

2 $$\begin{aligned} \mathbf {G}=\mathbf {M}\mathbf {M}^{{\mathrm{T}}}/(k/2), \end{aligned}$$

where $\mathbf {M}$ is the $n\times k$ allele content matrix with entries 1, $0,\, -1$ for genotypes 11, 12, 22, respectively, *n* is the total number of animals, *k* is the number of SNPs, and superscript T refers to the transpose of a matrix. The dominance genomic relationship matrix is:

3 $$\begin{aligned} \mathbf {D}=\mathbf {W}\mathbf {W}^{{\mathrm{T}}}/(k/4), \end{aligned}$$

where the $n\times k$ matrix $\mathbf {W}$ has entries 0, 1, 0 for genotypes 11, 12, 22, respectively. The genetic effects are correlated, i.e.

$$\begin{aligned} {\text {Var}}\left[ \begin{array}{c} \mathbf {u}_{p} \\ \mathbf {u}_{c} \\ \end{array} \right]= & {} \left[ \begin{array}{cc} \sigma _{u,p}^{2}\mathbf {G}_p &{} \sigma _{u,pc}\mathbf {G}_{pc} \\ \sigma _{u,pc}\mathbf {G}_{pc}^{{\mathrm{T}}} &{} \sigma _{u,c}^2\mathbf {G}_c \\ \end{array} \right] , \\ {\text {Var}}\left[ \begin{array}{c} \mathbf {v}_{p} \\ \mathbf {v}_{c} \\ \end{array} \right]= & {} \left[ \begin{array}{cc} \sigma _{v,p}^{2}\mathbf {D}_p &{} \sigma _{v,pc}\mathbf {D}_{pc} \\ \sigma _{v,pc}\mathbf {D}_{pc}^{{\mathrm{T}}} &{} \sigma _{v,c}^2\mathbf {D}_c \\ \end{array} \right] , \end{aligned}$$

with $\mathbf {G}_p$, $\mathbf {G}_{pc}$, and $\mathbf {G}_c$ being submatrices of matrix $\mathbf {G}$ in Eq. (2) for purebreds, between purebreds and crossbreds, and for crossbreds, respectively, and $\mathbf {D}_p$, $\mathbf {D}_{pc}$, and $\mathbf {D}_c$ being submatrices of matrix $\mathbf {D}$ in Eq. (3) for purebreds, between purebreds and crossbreds, and for crossbreds, respectively. The correlation between genotypic additive effects is $\rho _{u,pc}=\sigma _{u,pc}/\sqrt{\sigma ^2_{u,p}\sigma ^2_{u,c}}$ and the correlation between genotypic dominance effects is $\rho _{v,pc}=\sigma _{v,pc}/\sqrt{\sigma ^2_{v,p}\sigma ^2_{v,c}}$.

The variance–covariance matrices of the genetic effects defined above are not immediately suitable for fitting the model using standard genetic evaluation software. However, the variance–covariance structure of the genetic effects in the model can equivalently be described as:

$$\begin{aligned} {\text {Var}}\left[ \begin{array}{c} \mathbf {u}_{p} \\ \mathbf {u}_{p}^{\star } \\ \mathbf {u}_{c}^{\star } \\ \mathbf {u}_{c} \\ \end{array} \right] ={\varvec{\Sigma }}_u\otimes \mathbf {G},\ {\text {Var}}\left[ \begin{array}{c} \mathbf {v}_{p} \\ \mathbf {v}_{p}^{\star } \\ \mathbf {v}_{c}^{\star } \\ \mathbf {v}_{c} \\ \end{array} \right] ={\varvec{\Sigma }}_v\otimes \mathbf {D}, \end{aligned}$$

where $\mathbf {u}_{p}^{\star }$, $\mathbf {u}_{c}^{\star }$, $\mathbf {v}_{p}^{\star }$, and $\mathbf {v}_{c}^{\star }$ are vectors of artificial random effects, $\bigotimes$ is the Kronecker product, and ${\varvec{\Sigma }}_u$ and ${\varvec{\Sigma }}_v$ are $2\times 2$ variance–covariance matrices:

$$\begin{aligned} {\varvec{\Sigma }}_u= & {} \left[ \begin{array}{cc} \sigma _{u,p}^{2} &{} \sigma _{u,pc} \\ \sigma _{u,pc} &{} \sigma _{u,c}^{2} \\ \end{array} \right] \ , \\ {\varvec{\Sigma }}_v= & {} \left[ \begin{array}{cc} \sigma _{v,p}^{2} &{} \sigma _{v,pc} \\ \sigma _{v,pc} &{} \sigma _{v,c}^{2} \\ \end{array} \right] . \end{aligned}$$

This formulation of the variance–covariance structure of the genetic effects using artificial random effects and Kronecker products makes it possible to fit the model using standard genetic evaluation software. The model is similar to the model in Vitezica et al. [12] and Xiang et al. [13], except that only phenotypes of the crossbreds and the paternal line are included here, that the maternal population consists of crossbreds, and that inbreeding depression effects were not included in Vitezica et al. [12]. We name this model the genotypic model.

Inbreeding depression is incorporated in the model as a fixed effect, as in Xiang et al. [13], where the inbreeding coefficient of an individual is the frequency of homozygous loci for that individual, i.e. $\mathbf {f}_p=\mathbf {1}-\mathbf {W}_p\mathbf {1}/k$ and $\mathbf {f}_c=\mathbf {1}-\mathbf {W}_c\mathbf {1}/k$, where $\mathbf {1}$ is a vector with all elements being 1.

Note that for the three-way crossbred part of the model, additive and dominance effects for the three-way crossbred performance are assumed independent of the origin of the allele (sire line or crossbred sow population). An alternative would be a breed of origin effects model [9, 14], but this would require an accurate determination of the breed of origin of alleles, and also has been found to only to be beneficial in cases where traits have a combination of low heritability and low correlation between purebred and crossbred performance, and breeds are distantly related [15]. Therefore, this was not studied here.

#### Equivalent SNP effects model, and additive genetic variances and covariance

For the derivation of equations for genetic variances and covariances, and for EBV with associated accuracies that are presented in the following subsection, it is more suitable to express the genotypic effects $\mathbf {u}_{p}$, $\mathbf {u}_{c}$, $\mathbf {v}_{p}$ and $\mathbf {v}_{c}$ in model (1) equivalently as functions of SNP effects, $\mathbf {u}_{p}=\mathbf {M}_{p}\mathbf {a}_{p}$, $\mathbf {u}_{c}=\mathbf {M}_{c}\mathbf {a}_{c}$, $\mathbf {v}_{p}=\mathbf {W}_{p}\mathbf {d}_{p}$, $\mathbf {v}_{c}=\mathbf {W}_{c}\mathbf {d}_{c}$, where $\mathbf {a}_{p}$ and $\mathbf {a}_{c}$ are vectors of additive SNP effects, and $\mathbf {d}_{p}$ and $\mathbf {d}_{c}$ are vectors of dominance SNP effects. Here,

$$\begin{aligned} {\text {Var}}\left[ \begin{array}{c} \mathbf {a}_{p} \\ \mathbf {a}_{c} \\ \end{array} \right]= & {} \left[ \begin{array}{cc} \sigma _{a,p}^{2} &{} \sigma _{a,pc} \\ \sigma _{a,pc} &{} \sigma _{a,c}^{2} \\ \end{array} \right] \otimes \mathbf {I}, \\ {\text {Var}}\left[ \begin{array}{c} \mathbf {d}_{p} \\ \mathbf {d}_{c} \\ \end{array} \right]= & {} \left[ \begin{array}{cc} \sigma _{d,p}^{2} &{} \sigma _{d,pc} \\ \sigma _{d,pc} &{} \sigma _{d,c}^{2} \\ \end{array} \right] \otimes \mathbf {I}, \end{aligned}$$

where $\mathbf {I}$ is the identity matrix. Note that $\sigma ^2_{a,p}=\sigma ^2_{u,p}/(k/2)$, $\sigma _{a,c}^{2}=\sigma _{u,c}^{2}/(k/2)$, $\sigma _{a,pc}=\sigma _{u,pc}/(k/2)$, $\sigma ^2_{d,p}=\sigma ^2_{v,p}/(k/4)$, $\sigma _{d,c}^{2}=\sigma _{v,c}^{2}/(k/4)$ and $\sigma _{d,pc}=\sigma _{v,pc}/(k/4)$.

The inbreeding depression effects, $\mathbf {f}_{p}\eta _{p}$ and $\mathbf {f}_{c}\eta _{c}$ in model (1) are caused by directional dominance effects [13], which can be seen as follows. Consider the sire line part of the model, and the sum of the inbreeding depression effect and the genotypic dominance effect:

$$\begin{aligned} \mathbf {f}_{p}\eta _{p}+\mathbf {v}_p&=(\mathbf {1}-\mathbf {W}_{p}\mathbf {1}/k)\eta _{p}+\mathbf {W}_{p}\mathbf {d}_{p} \\&=\mathbf {1}\eta _{p}+\mathbf {W}_{p}((-\eta _{p}/k)\mathbf {1}+\mathbf {d}_{p}), \end{aligned}$$

then we see that $-\eta _{p}/k$ is the mean of the dominance SNP effects $(-\eta _{p}/k)\mathbf {1}+\mathbf {d}_{p}$, i.e. dominance SNP effects are directional. Assuming selection aims at increasing the trait, then $\eta _{p}$ would usually be negative, corresponding to inbreeding depression, and then the direction of the dominance SNP effects $-\eta _{p}/k$ would be positive. Similarly, for the three-way crossbred part, $-\eta _{c}/k$ is the mean of the directional dominance SNP effects $(-\eta _{c}/k)\mathbf {1}+\mathbf {d}_{c}$.

The classical additive genetic variance is not the parameter $\sigma ^2_{u,p}$, but according to Vitezica et al. [12] it is obtained from the vector of allele substitution effects and parameters $\sigma ^2_{a,p}$ and $\sigma ^2_{d,p}$ in the SNP effects model. Here, because the inbreeding depression effects in the model are caused by directional dominance effects, the vector of allele substitution effects is:

4 $$\begin{aligned} {\varvec{\alpha }}_p=\mathbf {a}_{p}+((-\eta _{p}/k)\mathbf {1}+\mathbf {d}_{p})*(\mathbf {q}_{p}-\mathbf {p}_{p}), \end{aligned}$$

where $\mathbf {p}_{p}$ and $\mathbf {q}_{p}$ are vectors of frequencies of the first and second allele, respectively, and * denotes elementwise multiplication. Making the standard assumptions of linkage equilibrium and uncorrelated marker effects across loci (see e.g. [12]), and letting superscript *j* denote the* j*th element of vectors, the resulting expression for the additive genetic variance is:

5 $$\begin{aligned} \sigma ^2_{g,p}=&\sum _j 2p^j_{p} q^j_{p} {\text {E}}[(\alpha ^p_j)^2] \\ \nonumber =&\sum _j 2p^j_{p} q^j_{p} \sigma ^2_{a,p} \\&+\sum _j 2p^j_{p} q^j_{p} (q^j_{p}-p^j_{p} )^2(\sigma ^2_{d,p}+\eta ^2_{p}/k^2), \nonumber \end{aligned}$$

since ${\text {E}}[(\alpha ^j_p)^2]=\sigma ^2_{a,p}+(q^j_{p}-p^j_{p} )^2(\eta ^2_{p}/k^2+\sigma ^2_{d,p})$. The expression in Eq. (5) differs from the corresponding equation in Xiang et al. [13], because they overlooked the contribution from $\eta ^2_p/k^2$ to the equation. Following Xiang et al. [13], here we name this the additive genetic variance for purebred performance (mating within sire line).

The additive genetic variance for crossbred performance (mating sire line with sows from another population) is according to Xiang et al. [13] obtained from the vector of allele substitution effects for sire line boars when mated to the specific population. However, compared to Xiang et al. [13], one difference is that the population of crossbred sows cannot be assumed to be in Hardy–Weinberg equilibrium, and the usual equation for the allele substitution effect does not apply. Instead we use Eq. (11) in Falconer [16], which says that the allele substitution effect equals $a+d(q-p)(1-F)/(1+F)$, where *F* measures departure from Hardy Weinberg equilibrium, and equals $F=1-h/(2pq)$ with *h* being the frequency of the heterozygous genotypes (with the definition that $F=0$ when $p=0$ or $q=0$). Using $(1-F)/(1+F)=h/(4pq-h)$, the vector of allele substitution effects can therefore be expressed as:

6 $$\begin{aligned} {\varvec{\alpha }}_c=\mathbf {a}_{c}+((-\eta _{c}/k)\mathbf {1}+\mathbf {d}_{c})*\mathbf {r}_{cs}, \end{aligned}$$

with

7 $$\begin{aligned} \mathbf {r}_{cs}=\frac{(\mathbf {q}_{cs}-\mathbf {p}_{cs})*\mathbf {h}_{cs}}{4\mathbf {p}_{cs}*\mathbf {q}_{cs}-\mathbf {h}_{cs}}, \end{aligned}$$

where $\mathbf {p}_{cs}$ and $\mathbf {q}_{cs}$ are vectors of frequencies of the first and second allele in crossbred sows, respectively, $\mathbf {h}_{cs}$ is a vector of frequencies of heterozygous genotypes in crossbred sows, and * and fraction in Eq. (7) denote elementwise multiplication and division, respectively. Note that for elements with $p^j_{cs}=0$ or $q^j_{cs}=0$, the fraction in Eq. (7) is to be understood as $r^j_{cs}=(q^j_{cs}-p^j_{cs})$ to avoid the problem of division by zero, and this convention also applies in Eqs. (8), (9), (13) and (14) below. The resulting expression for the additive genetic variance for crossbred performance of sire line boars (mating with crossbred sows) is:

8 $$\begin{aligned} \sigma _{g,c}^2=&\sum _j 2p_{p}^j q_{p}^j\sigma _{a,c}^2 \\&+\sum _j 2p_{p}^j q_{p}^j(r_{cs}^j)^2(\sigma _{d,c}^2+\eta _{c}^2/k^2), \nonumber \end{aligned}$$

where $r^j_{cs}$ is the $j{\text{th}}$ element of the vector defined in Eq. (7).

The additive genetic covariance between purebred and crossbred performances is according to Xiang et al. [13] obtained from covariances between allele substitution effects. Here, this becomes:

9 $$\begin{aligned} \sigma _{g,pc}=&\sum _j 2 p_{p}^j q_{p}^j {\text {E}}[\alpha ^j_p\alpha ^j_c]\\ \nonumber =&\sum _j 2 p_{p}^j q_{p}^j\sigma _{a,pc} \\ \nonumber&+\sum _j 2 p_{p}^j q_{p}^j (q_{p}^j-p_{p}^j )r_{cs}^j (\sigma _{d,pc}+\eta _{p}\eta _{c}/k^2). \end{aligned}$$

The genetic correlation between purebred and crossbred performances then equals

10 $$\begin{aligned} \rho _{g,pc}=\sigma _{g,pc}/\sqrt{\sigma _{g,p}^2\sigma _{g,c}^2}, \end{aligned}$$

where $\sigma ^2_{g,p}$, $\sigma ^2_{g,c}$, and $\sigma _{g,pc}$ are defined in Eqs. (5), (8) and (9), respectively.

### Breeding values for purebred and crossbred performances

Breeding values (BV) of sire line boars for purebred performance are obtained from the allele substitution effects shown in Eq. (4) as ${\mathbf {BV}}_p=\mathbf {Z}_{p}(\mathbf {a}_{p}+((-\eta _{p}/k)\mathbf {1}+\mathbf {d}_{p})*(\mathbf {q}_{p}-\mathbf {p}_{p}))$, where $\mathbf {Z}_{p}=\mathbf {M}_{p}-\mathbf {1}(2\mathbf {p}_{p}-\mathbf {1})^{{\mathrm{T}}}$. EBV are obtained from estimated effects, ${\hat{\mathbf {a}}}_{p}$, ${\hat{\mathbf {d}}}_{p}$, ${\hat{\eta }}_{p}$ in the SNP effects model,

11 $$\begin{aligned} {\mathbf {EBV}}_p=\mathbf {Z}_{p}({\widehat{\mathbf {a}}}_{p}+((-{\hat{\eta }}_{p}/k)\mathbf {1}+{\widehat{\mathbf {d}}}_{p})*(\mathbf {q}_{p}-\mathbf {p}_{p})). \end{aligned}$$

Alternatively, EBV can be obtained from the estimated effects, ${\hat{\mathbf {u}}}_{p}$, ${\hat{\mathbf {v}}}_{p}$, ${\hat{\eta }}_{p}$, in the genotypic model by backsolving as described in the following. Based on the fact that estimated effects satisfy ${\widehat{\mathbf {a}}}_{p}=(k/2)^{-1}\mathbf {M}_{p}^{{\mathrm{T}}}(\mathbf {G}_{p})^{-1}{\widehat{\mathbf {u}}}_{p}$ and ${\widehat{\mathbf {d}}}_{p}=(k/4)^{-1}\mathbf {W}_{p}^{{\mathrm{T}}}(\mathbf {D}_{p})^{-1}{\widehat{\mathbf {v}}}_{p}$, which follows from combining Eqs. 5 and 6 in Stranden and Garrick [17], we obtain:

12 $$\begin{aligned} {\mathbf {EBV}}_p=\, & {} \mathbf {Z}_{p}\mathbf {M}_{p}^{{\mathrm{T}}}(\mathbf {G}_{p})^{-1}{\widehat{\mathbf {u}}}_{p}/(k/2) \\&+\mathbf {Z}_{p}((-{\hat{\eta }}_{p}\mathbf {1}/4+\mathbf {W}_{p}^{{\mathrm{T}}}(\mathbf {D}_{p})^{-1}{\widehat{\mathbf {v}}}_{p})*(\mathbf {q}_{p}-\mathbf {p}_{p}))/(k/4). \nonumber \end{aligned}$$

Accuracy of EBV, $acc_i=\text {cor}(BV_i,EBV_i)$, can be obtained from the prediction error variance (PEV) as $acc_{p,i} = 1-\sqrt{PEV_{p,i}/{\text {Var}}(BV_{p,i})}$, where ${\text {Var}}(BV_{p,i})=\,(\mathbf {Z}_{p}\mathbf {Z}_{p}^{{\mathrm{T}}})_{ii}\sigma ^2_{a,p}+(\mathbf {Z}_{p}{\varvec{\Delta }}^2_p\mathbf {Z}_{p}^{{\mathrm{T}}})_{ii}(\sigma ^2_{d,p}+\eta _p^2/k^2)$, with ${\varvec{\Delta }}_p$ being a diagonal matrix with elements $\mathbf {q}_{p}-\mathbf {p}_{p}$. Details on how to compute PEV for the SNP effects model are in Appendix. It is unclear how to compute this PEV for the genotypic model.

Breeding values of sire line boars for three-way crossbred performance are obtained from the allele substitution effects of sire line boars for crossbred performance as shown in Eq. (6), and can therefore be expressed as ${\mathbf {BV}}_c=\mathbf {Z}_{p}(\mathbf {a}_{c}+((\eta _{c}/k)\mathbf {1}+\mathbf {d}_{c})*\mathbf {r}_{cs})$, where $\mathbf {r}_{cs}$ is defined in (7). Thus, from the SNP effects model, EBV for three-way crossbred performance of sire line boars are equal to:

13 $$\begin{aligned} {\mathbf {EBV}}_c=\mathbf {Z}_{p}({\widehat{\mathbf {a}}}_{c}+((-{\hat{\eta }}_{c}/k)\mathbf {1}+{\widehat{\mathbf {d}}}_{c})*\mathbf {r}_{cs}), \end{aligned}$$

and accuracies can be obtained from PEV as $acc_{c,i}=1-\sqrt{PEV_{c,i}/{\text {Var}}(BV_{c,i})}$, where ${\text {Var}}(BV_{c,i})=(\mathbf {Z}_{p}\mathbf {Z}_{p}^{{\mathrm{T}}})_{ii}\sigma ^2_{a,c}+(\mathbf {Z}_{p}{\varvec{\Delta }}^2_c\mathbf {Z}_{p}^{{\mathrm{T}}})_{ii}(\sigma ^2_{d,c}+\eta _c^2/k^2)$, with ${\varvec{\Delta }}_c$ being a diagonal matrix with elements $\mathbf {r}_{cs}$, and with $PEV_c$ computed as explained in Appendix. Alternatively, based on the fact that the estimated effects satisfy Eqs. ${\widehat{\mathbf {a}}}_{c}=(k/2)^{-1}\mathbf {M}_{c}^{{\mathrm{T}}}(\mathbf {G}_{c})^{-1}{\widehat{\mathbf {u}}}_{c}$ and ${\widehat{\mathbf {d}}}_{c}=(k/4)^{-1}\mathbf {W}_{c}^{{\mathrm{T}}}(\mathbf {D}_{c})^{-1}{\widehat{\mathbf {v}}}_{c}$, which again are based on Eqs. 5 and 6 of Stranden and Garrick[17], we obtain the following equation for EBV based on the genotypic model:

14 $$\begin{aligned} {\mathbf {EBV}}_c= & {} \mathbf {Z}_{p}\mathbf {M}_{c}^{{\mathrm{T}}}(\mathbf {G}_{c})^{-1}{\widehat{\mathbf {u}}}_{c}/(k/2) \\&+\mathbf {Z}_{p}\left( (-{\hat{\eta }}_{c}\mathbf {1}/4+\mathbf {W}_{c}^{{\mathrm{T}}}(\mathbf {D}_{c})^{-1}{\widehat{\mathbf {v}}}_{c})*\mathbf {r}_{cs} \right) /(k/4). \nonumber \end{aligned}$$

### Data

The data set consisted of Danbred Duroc and Danbred DLY pigs reared in the same time period at the test station Bøgildgaard in Denmark, and measured for phenotypes on production traits. All pigs had known pedigree, and sires of both the Duroc and the DLY populations were active breeding boars in the DanBred Duroc population (139 and 140 sires for the Duroc and DLY populations, respectively, with 61 overlapping between the two populations), which creates a genetic relationship between the Duroc and the DLY pigs used in this study. The Duroc pigs were part of the routine breeding program in which owners of nucleus herds select up to three male pigs from litters with a high index (average of indices for parents) for entering the test station. The DLY pigs were produced at a commercial sow herd, for which 698 litters of DLY pigs were produced with the intention of having four pigs (two males and two females) from each litter in the test. The Duroc pigs came from 13 nucleus herds, and the DLY pigs came from one sow herd, resulting in a low biosecurity level at the test station due to the mixing of pigs from many herds with different disease status. At the test station, feed was given ad libitum in feeders with individual recordings on feed intake and each pen was planned to contain 14 pigs. During the study, changes of feed composition at the test station were made twice, but in all cases the feed was optimised according to Danish nutrient recommendations [18]. The experiment was designed to have phenotypes and genotypes on all these animals, but for various reasons this was not completely achieved. Only animals which had both phenotypes and genotypes (after quality control described below) were analysed here, and the resulting data set contained 2595 Duroc and 2426 DLY pigs that entered the test station between 18 June 2014 and 17 August 2015, and that had their ultra-sound backfat thickness recorded between 5 August 2014 and 5 October 2015. In addition, most animals had their Duroc sire genotyped. In this study, all the Duroc pigs were males, whereas the DLY pigs consisted of approximately 50% males and 50% females.

The pigs were genotyped with either the 8.5K GGP-Porcine LD Illumina Bead SNP array or the GeneSeek Genomic Profiler (GGP) Porcine HD 70K SNP array (almost all DLY pigs and nearly two thirds of the Duroc pigs were genotyped with the 8.5K array). For simplicity, and to avoid issues with effects of imputation errors, only SNPs that overlapped between the two SNP arrays were used here. Animals with parentage error due to the Duroc sire (fraction of Mendelian mismatches between animal and sire higher than 2%) were removed, and otherwise genotypes with such mismatches were declared missing. The SNPs were quality controlled using the following criteria: SNPs with a call-rate lower than 90% or with a minor allele frequency (MAF) lower than 0.01 were excluded. After quality control, 6316 SNPs were retained. The genotype of an animal was only retained if the call rate was higher than 90%. Following this quality control, 1.2% of genotypes were missing, and these were imputed using Fimpute [19].

The following four traits were analysed: ultra-sound recorded backfat thickness (BF), overall conformation score (CONF), average daily gain (ADG), and feed conversion ratio (FCR). BF was measured as the average of ultrasound measurements in mm at four positions of the back, recorded at the end of the test period, CONF was measured as a score ranging from 2 to 4 at the end of test period, ADG was total weight gain in kg during the test period divided by number of days of the test period, and FCR was total feed intake in kg divided by total weight gain in kg during the test period. One animal with an unusually low end weight of 43 kg was removed from the study, and for one animal that had an unusually large feed intake given the weight gain, this feed intake was changed to missing. A summary of the data is in Table 1.

**Table 1:** Summary statistics of the phenotypes measured on Duroc and DLY pigs

	N	Min	Median	Mean	Max	St. dev.
Duroc						
BF	2595	4.50	7.50	7.40	11.50	0.74
CONF	2595	2	3	3.01	4	0.66
ADG	2595	0.61	1.13	1.12	1.56	0.13
FCR	2586	1.50	2.01	2.11	3.42	0.19
Start wgt	2595	28.0	31.0	32.9	62.0	5.7
End wgt	2595	70.0	92.0	91.8	122.0	10.4
DLY						
BF	2425	4.25	8.00	7.91	12.50	0.96
CONF	2425	2	3	3.11	4	0.62
ADG	2425	0.57	1.09	1.08	1.54	0.14
FCR	2420	1.60	2.17	2.18	4.00	0.19
Start wgt	2425	28.0	30.0	31.3	58.0	3.6
End wgt	2425	60.0	87.0	86.8	124.0	11.7

*BF* ultra-sound recorded backfat thickness in mm, *CONF* overall conformation score, *ADG* average daily gain in kg, *FCR* feed conversion ratio

### Data analysis

For the analysis of traits BF, CONF, ADG or FCR observed on the Duroc sire line and DLY crossbreds, as presented in the subsection on data, we used the bivariate model (1), where vectors $\mathbf {y}_{p}$ and $\mathbf {y}_c$ contained phenotypes (BF, CONF, ADG or FCR) on Duroc and DLY respectively, vectors ${\varvec{\beta }}_{p}$ and ${\varvec{\beta }}_c$ contained section-year-season effects, sex effects (only ${\varvec{\beta }}_c$), and a regression on weight at time of recording (for BF and CONF), or on start weight (for ADG and FCR), matrices $\mathbf {X}_{p}$ and $\mathbf {X}_{c}$ relate animals with phenotypes to section-year-season’s and sexes (only for ${\varvec{\beta }}_c$) and contain a column with weights at time of recording (for BF and CONF) or start weight (for ADG and FCR), and the remaining notation is defined in the subsection entitled Combined purebred and crossbred model.

The allele frequencies of sire line and crossbred sows and heterozygote genotype frequencies in crossbred sows all enter into Eqs. (5), (8) and (9) for genetic variances of and covariance between purebred and crossbred performances. For this study, we computed these allele frequencies as follows: the sire line allele frequencies were the observed allele frequencies of the purebred Duroc in this study, whereas the allele and genotype frequencies in LY were computed from observed allele frequencies on genotypes of 9021 Danbred Landrace and 8760 Danbred Yorkshire animals born in 2014, i.e. $p_{cs}^j=(p_L^j+p_Y^j)/2$ and $p^j_{cs,12}=p^j_L q^j_Y+ p^j_Y q^j_L$, where $p^j_L=1-q^j_L$ and $p^j_Y=1-q^j_Y$ were allele frequencies for Landrace and Yorkshire pigs, respectively, born in 2014. Parameter estimates (with associated standard errors) and estimated effects were obtained from the genotypic model, using restricted maximum likelihood and best linear unbiased estimation implemented in the DMUAI module in the software DMU [20]. For all traits, convergence of the AI-REML algorithm for parameter estimation was slow, which indicated possible problems of lack of convergence. To assess this, investigations with different starting values were made to confirm that the resulting parameter estimates did not depend on starting values for the algorithm. Genetic variances, covariances, correlations, and EBV were estimated using Eqs. (5), (8), (9), (10), (12) and (14), and heritabilities were estimated using $h^2_p=\sigma ^2_{g,p}/(\sigma ^2_{a,p}\bar{\delta G_p}+ \sigma ^2_{d,p}\bar{\delta D_p}+ \sigma ^2_{l,p}+ \sigma ^2_{e,p})$ and $h^2_c=\sigma ^2_{g,c}/(\sigma ^2_{a,c}\bar{\delta G_c}+ \sigma ^2_{d,c}\bar{\delta D_c}+ \sigma ^2_{l,c}+\sigma ^2_{e,c})$, where $\bar{\delta G_p}$, $\bar{\delta D_p}$ $\bar{\delta G_c}$ and $\bar{\delta D_c}$ were averages of diagonal elements in matrices $\mathbf {G}_p$, $\mathbf {D}_p$, $\mathbf {G}_c$, and $\mathbf {D}_c$, respectively. Standard errors on estimates of parameters were computed using the delta-method implemented in the contributed R package car, version 2.1-5 [21]. Accuracies of EBV were computed based on PEV, which were computed as explained in Appendix based on the SNP effects model using a method in the DMU4 module of the software DMU release 5.4 [20] that returns the coefficient matrix of the mixed models equations.

## Results

**Table 2:** Best linear unbiased estimates of inbreeding depression parameters and estimates of variance and covariance parameters

	BF	CONF	ADG	FCR
$\eta _{p}$	1.28 (0.69)	− 0.21 (0.93)	− 0.46 (0.18)	0.18 (0.23)
$\eta _{c}$	0.50 (1.26)	0.22 (1.14)	− 0.26 (0.23)	− 0.07 (0.30)
$\sigma ^2_{u,p}$	0.151 (0.017)	0.118 (0.021)	0.0016 (0.0005)	0.0033 (0.0010)
$\sigma ^2_{u,c}$	0.227 (0.030)	0.067 (0.019)	0.0045 (0.0009)	0.0046 (0.0013)
$\sigma _{u,pc}$	0.182 (0.019)	0.075 (0.016)	0.0024 (0.0006)	0.0039 (0.0009)
$\rho _{u,pc}$	0.98 (0.07)	0.84 (0.17)	0.88 (0.21)	0.99 (0.21)
$\sigma ^2_{v,p}$	0.005 (0.008)	0.002 (0.015)	0.0012 (0.0006)	0.0023 (0.0010)
$\sigma ^2_{v,c}$	0.009 (0.021)	0.001 (0.017)	0.0004 (0.0007)	0.0002 (0.0011)
$\sigma _{v,pc}$	0.007 (0.012)	0.001 (0.015)	− 0.0005 (0.0006)	0.0007 (0.0010)
$\rho _{v,pc}$	1.00 (2.34)	1.00 (15.91)	− 0.71 (1.01)	1.00 (3.01)
$\sigma ^2_{l,p}$	0.029 (0.009)	0.007 (0.019)	0.0014 (0.0006)	0.0006 (0.0010)
$\sigma ^2_{l,c}$	0.028 (0.010)	0.008 (0.008)	0.0011 (0.0004)	0.0029 (0.0006)
$\sigma ^2_{e,p}$	0.145 (0.010)	0.360 (0.021)	0.0105 (0.0007)	0.0184 (0.0012)
$\sigma ^2_{e,c}$	0.303 (0.015)	0.320 (0.014)	0.0106 (0.0005)	0.0191 (0.0009)

Best linear unbiased estimates of inbreeding depression parameters and estimates of variance and covariance parameters with associated standard errors (in brackets) for the four traits

*BF* ultra-sound recorded backfat thickness, *CONF* overall conformation score, *ADG* average daily gain, *FCR* feed conversion ratio

Table 2 shows the best linear unbiased estimates of inbreeding depression parameters and estimates of variance and covariance parameters with associated standard errors (in brackets) for the four traits. Estimates of correlations between genotypic additive effects for purebred and crossbred performances in Table 2, $\rho _{u,pc}$, (with associated standard errors) were equal to 0.98 (0.07), 0.84 (0.17), 0.88 (0.21), 0.99 (0.21) for BF, CONF, ADG, FCR, respectively, and estimates of correlations between genotypic dominance effects for purebred and crossbred performances, $\rho _{v,pc}$, (with associated standard errors) were equal to 1.00 (2.34), 1.00 (15.91), − 0.71 (1.01), 1.00 (3.01) for BF, CONF, ADG, FCR, respectively. Furthermore, the $-2$*log-likelihood ratio statistic for testing the model without dominance effects was 1.08, 0.02, 6.17, 7.19 for BF, CONF, ADG, FCR, respectively, and these were evaluated against a $\chi ^2(3)$-distribution, since three parameters (two variance parameters and one covariance parameter) were removed from the model. Since the $95\%$ quantile of a $\chi ^2(3)$-distribution is 7.81, the dominance effects were negligible for CONF, but not for the other three traits, although we cannot reject the hypothesis of no dominance effects for these traits.

The results in Table 2 show that for purebred Duroc pigs, the estimated inbreeding depression per unit of inbreeding, $\eta _{p}$ was negative for ADG, and statistically significantly different from zero. A negative value of $\eta _{p}$ means inbreeding depression for ADG. For DLY, there was also a negative inbreeding depression effect for ADG, although it was not statistically significantly different from zero. The estimated $\eta _{p}$ and $\eta _{c}$ for BF were both positive (corresponding to inbreeding depression since direction of selection is to reduce BF), but neither was statistically significantly different from zero. For the other two traits, the estimates of $\eta _{p}$ and $\eta _{c}$ were both small when the standard error estimate was taken into account, and they also had conflicting signs between Duroc and DLY.

Based on estimates of model parameters and allele frequencies, genetic parameters were computed using Eqs. (5), (8) and (9). The resulting estimates of additive genetic variances, covariance and correlation (with associated standard errors) for purebred and crossbred performances for each trait are in Table 3. For BF, ADG, and FCR, the genetic variance was smaller for purebred performance than for crossbred performance, whereas the opposite was observed for CONF. The difference between estimates of additive genetic variances for purebred and crossbred performances was most pronounced for ADG, for which $\sigma ^2_{g,c}$ was more than two times larger than $\sigma ^2_{g,p}$. Estimates of additive genetic correlations between purebred and crossbred performances ranged from 0.75 for ADG to 0.96 for BF.

**Table 3:** Additive genetic parameters and heritabilities

	BF	CONF	ADG	FCR
$\sigma ^2_{g,p}$	0.085 (0.009)	0.067 (0.011)	0.0012 (0.0003)	0.0023 (0.0005)
$\sigma _{g,c}^{2}$	0.131 (0.015)	0.038 (0.009)	0.0027 (0.0004)	0.0027 (0.0006)
$\sigma _{g,pc}$	0.102 (0.010)	0.042 (0.009)	0.0013 (0.0003)	0.0022 (0.0005)
$\rho _{g,pc}$	0.96 (0.07)	0.83 (0.16)	0.75 (0.17)	0.87 (0.18)
$h^2_p$	0.22 (0.01)	0.12 (0.02)	0.08 (0.02)	0.09 (0.02)
$h^2_c$	0.21 (0.02)	0.09 (0.02)	0.15 (0.02)	0.10 (0.02)

Additive genetic parameters for the four traits computed using Eqs. (5), (8) and (9) from model parameter estimates in Table 2. Variance for purebred performance: $\sigma ^2_{g,p}$, Variance for crossbred performance: $\sigma ^2_{g,c}$, covariance between purebred and crossbred performances: $\sigma _{g,pc}$, correlation between purebred and crossbred performances: $\rho _{g,pc}$, and heritabilities, $h^2_p=\sigma ^2_{g,p}/(\sigma ^2_{a,p}\bar{\delta G}_p+ \sigma ^2_{d,p}\bar{\delta D_p}+ \sigma ^2_{l,p}+ \sigma ^2_{e,p})$ and $h^2_c=\sigma ^2_{g,c}/(\sigma ^2_{a,c}\bar{\delta G_c}+ \sigma ^2_{d,c}\bar{\delta D_c}+ \sigma ^2_{l,c}+\sigma ^2_{e,c})$ where $\bar{\delta G_p},\, \bar{\delta D_p}$ $\bar{\delta G_c}$ and $\bar{\delta D_c}$ are averages of diagonal elements in matrices $\mathbf {G}_p$, $\mathbf {D}_p$, $\mathbf {G}_c$, and $\mathbf {D}_c$, respectively

*BF* ultra-sound recorded backfat thickness, *CONF* overall conformation score, *ADG* average daily gain, *FCR* feed conversion ratio

**Fig. 1:**
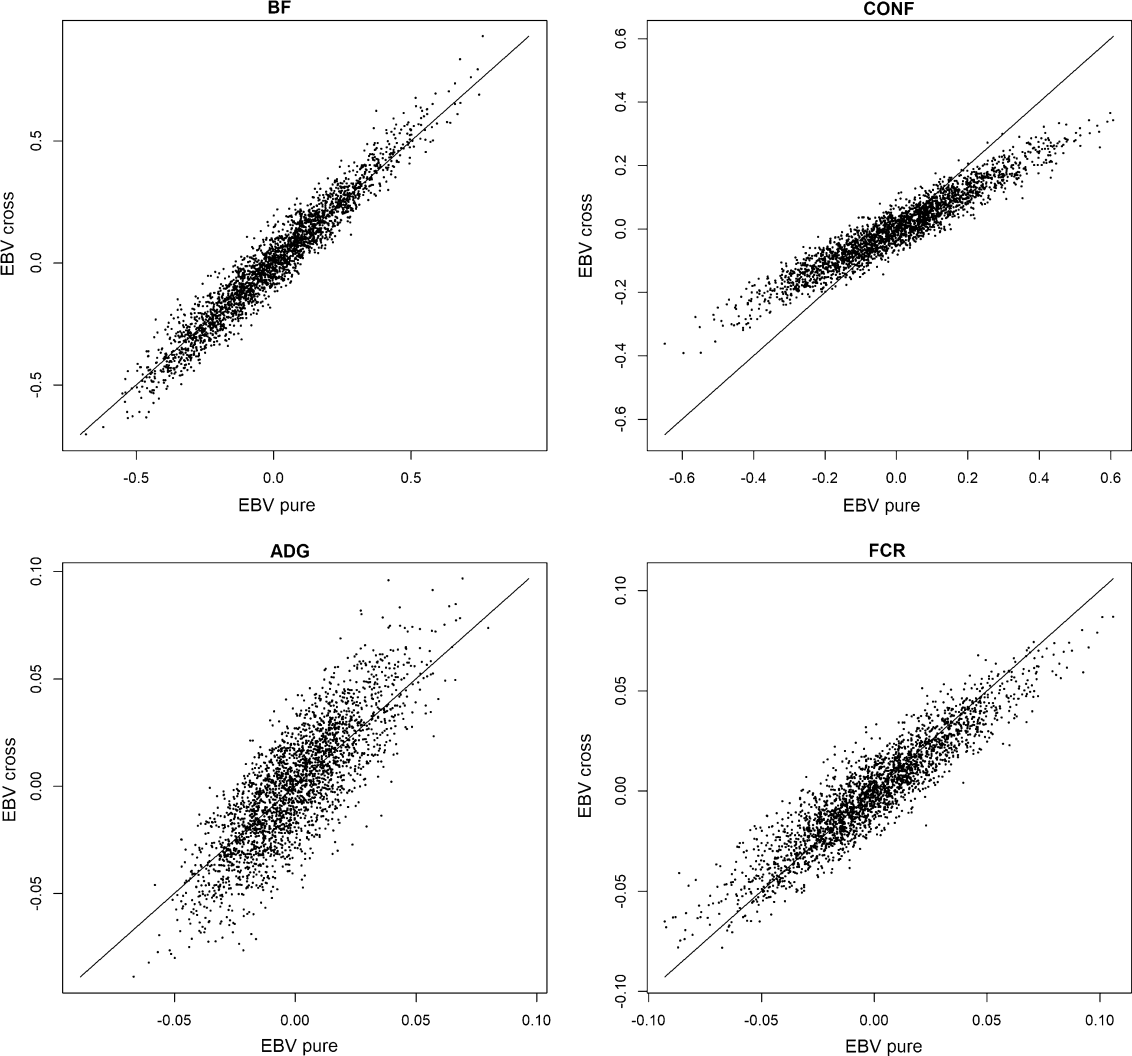
EBV for purebred and crossbred performances. EBV for purebred and crossbred performances on Duroc boars. *BF* ultra-sound recorded backfat thickness, *CONF* overall conformation score, *ADG* average daily gain, *FCR* feed conversion ratio

EBV of Duroc boars for purebred and crossbred performances are in Fig. 1, which shows that there was a strong positive association between EBV for purebred and crossbred performances when the additive genetic correlation $\rho _{g,pc}$ was large (e.g. trait BF), and that the association was weaker when the additive genetic correlation was weaker (e.g. trait ADG). Figure 1 also shows that the variability of EBV for crossbred performance compared to variability for purebred performance was smaller for CONF, similar for FCR and BF, and larger for ADG. This pattern is consistent with the pattern of additive genetic variances seen in Table 3. Equations (11) and (13) for EBV both use allele frequencies for Duroc boars to center matrix $\mathbf {Z}_p$ and, therefore, these EBV are relative to the population of Duroc boars. As a result, the EBV are centered around zero in Fig. 1.

**Fig. 2:**
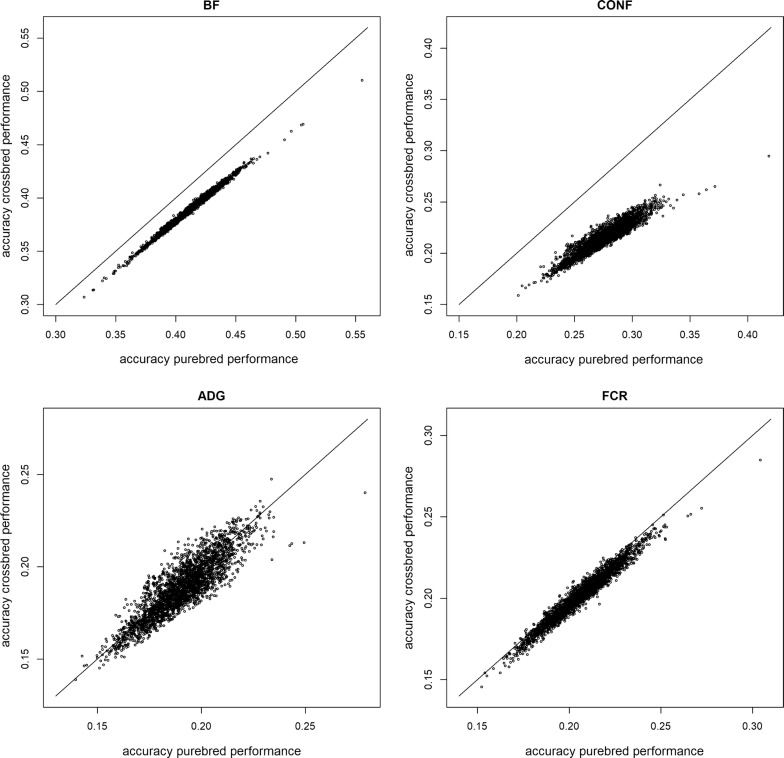
Accuracies of EBV for purebred and crossbred performances. Accuracies of EBV for purebred and crossbred performances on Duroc boars. *BF* ultra-sound recorded backfat thickness, *CONF* overall conformation score, *ADG* average daily gain, *FCR* feed conversion ratio

Accuracies of EBV for purebred and crossbred performances of Duroc boars are in Fig. 2, which shows a positive relationship between accuracies for purebred and crossbred performances. For BF, CONF and FCR, accuracies were higher for purebred performance than for crossbred performances, whereas the opposite was observed for ADG. The amount of information on relatives is an important parameter for accuracies. For the data set investigated here, seven of the Duroc boars had Duroc offspring with records, but none had DLY offspring with records. In general, these seven boars with Duroc offspring had the highest accuracies of EBV for purebred performance (for BF, CONF, and AGD the top five boars in terms of accuracy for purebred performance all had Duroc offspring, for ADG four of the top five boars in terms of accuracy had Duroc offspring), and also had high accuracies for crossbred performance (accuracy gained from the correlated purebred performance with high accuracy).

## Discussion

We present a model for genomic evaluation for a terminal three-way crossbreeding system when information is available on the purebred sire line and three-way crossbred pigs. This model is a modification of the models studied in Vitezica et al. [12] and Xiang et al. [13], accounts for both additive and dominance genotypic effects, and provides flexibility through genetic correlations for these effects between purebreds and crossbreds. We also present an equivalent SNP effects model, and equations were derived for additive genetic parameters as functions of parameters in the model and allele frequencies, for EBV for purebred and crossbred performances as functions of estimated effects in the model and allele frequencies, and for accuracies of EBV as functions of allele frequencies and submatrices of the inverse of the coefficient matrix of the mixed model equations. The methods were used to analyse a data set of BF, CONF, ADG and FCR measured on Duroc and DLY pigs.

The model in this paper includes both additive and dominance genotypic effects, which are allowed to differ between purebred and crossbred animals, but correlated. The estimates of correlation between additive genotypic effects, $\rho _{a,pc}$, were large for all traits, but in all cases lower than 1. In this study, the environments were the same for purebreds and crossbreds, and based on these results, it is possible to speculate whether the functional additive genetic effects are the same in purebreds and crossbreds. However, the markers included in the model are probably not the causal loci, and differences in linkage equilibrium between markers and causal loci between lines would result in a correlation lower than 1, even if the functional additive genetic effects at causal loci were the same. For BF, CONF and FCR, correlations between dominance genotypic effects, $\rho _{v,pc}$, were about 1, whereas for ADG it was − 0.71. The negative correlation estimate for ADG is puzzling, although it has to be taken into account that the standard error on the estimate was large.

For BF, FCR and ADG, the additive genetic variance was smaller for purebred performance than for crossbred performance. This may be related to a difference in how purebred and crossbred pigs were selected for entering the test station: crossbred pigs were selected randomly from litters (about 4 pigs from each litter) from sires with a low, medium or high index, whereas purebred pigs were selected among litter mates (1–2 pigs from a litter) from litters with a high index by the owners of the nucleus herds. The owners of the nucleus herds had clear economic interests in having the best performing boars at the test station. Therefore, the selection of purebred pigs entering the test station was not random, and very likely this selection was not independent of the phenotypic records observed in the study, which likely resulted in a reduced genetic variation, in particular for ADG.

Guo et al. [22] analysed BF and ADG in DanBred Duroc using a model with additive and dominance genetic effects, and obtained estimates of additive genetic variances equal to 0.342 mm$^2$ for BF and 0.000828 kg$^2$ for ADG. This estimate of the additive genetic variance for BF was about four times larger than the estimate reported in our paper, which is a substantial difference. However, the estimate of the residual variance in Guo et al. [22] was about twice as large as that found here, and they used data over a longer time span, which were recorded both at the test station Bøgildgaard and at a number of nucleus herds. They also did not correct for weight at recording. All together, this results in a larger variability of their measures, and, therefore, the difference in genetic variance may make sense. Regarding the estimate of additive genetic variance parameter for ADG, the estimate in Guo et al. [22] was substantially smaller than that reported here, which may make sense since their estimate of residual variance was also much smaller than ours and since the trait ADG in Guo et al. [22] was standardised to the weight interval 30 to 100 kg.

The estimated additive genetic correlations between purebred and crossbred performances, $\rho _{g,pc}$, in our study ranged from 0.75 for ADG to 0.96 for BF, which in general are larger than averages of estimates for similar traits reported in the literature (see Figure 3 in the review paper by Wientjes and Calus [23]). In our study, the crossbred and the purebred animals were located in the same production unit at the same time under the same conditions. Thus, one reason for the low correlations reported in other studies could be the difference in environments between purebred and crossbred animals. Another reason may be that the differences in allele frequencies between the studied populations were larger in the other studies than in ours.

ADG was the only trait for which there was a statistically significant inbreeding depression effect, and only in the Duroc population. This is not surprising since inbreeding depression is particularly important for traits that have been under strong selection [24].

For the traits BF, CONF and FCR, accuracies of EBV for Duroc boars for purebred performance were higher than those for crossbred performance, while they were simlar for ADG. Several factors influence accuracy, such as information on relatives and heritability. As mentioned in the Results section, seven of these Duroc boars had Duroc offspring, which increases the accuracies of purebred performance for these boars. More generally, the Duroc boars all had own records on purebred performance, and not on crossbred performance, which increases the accuracies of EBV for purebred performances for all traits. In terms of heritability (see Table 2), estimates for BF, CONF and FCR did not differ substantially between purebred performance and crossbred performance, but for ADG the estimate was higher for crossbred performance than for purebred performance. Since high heritability implies high accuracy, the pattern in heritability estimates combined with the fact that these boars had own records on purebred performance, results in the observed pattern of accuracies of EBV.

Results on predictive performance based on splitting the data set into training data and validation data were reported by Xiang et al. [13], and they showed that including inbreeding depression in the model improved predictive performance of total genotypic effects, whereas including dominance effects did not. For the data set in our paper, an investigation of predictive performance for models with or without inbreeding depression and dominance effects was also attempted, but only one of the differences between models was statistically significant, and the pattern of results was very inconsistent across traits and purebreds and crossbreds; see Additional file 1. We believe that the small size of the data set could be the cause of these inconsistent results.

Although our model contains four genetic effects, it was feasible to fit the model within reasonable time and with reasonable use of computer memory because the data set analysed was relatively small, both in terms of number of animals and number of SNPs. For data sets with a large number of animals, both computing time and memory use may become a challenge for the genotypic model, due to the size and non-sparsity of the system of mixed model equations. For the SNP effects model, we computed accuracies of EBV by inverting the coefficient matrix of the mixed model equations, which was feasible for the data set investigated here, since the number of SNPs was small. For a data set with large number of SNPs, an approximative method, such as that in Tier and Meyer [25] would need to be derived and implemented.

In general, marker genotypes may not capture all the genetic effects, and it is therefore common practice to add a residual polygenic effect with pedigree-structure into the genomic model, see e.g. Mrode [6]. For the model considered here, four residual polygenic effects would need to be added to the model, i.e. additive and dominance effects for both the sire line and crossbreds, which would imply a larger computational burden of parameter estimation and prediction of effects. In addition, different types of SNP arrays are often used. In this study, only the SNPs that overlapped between two SNP arrays were used in the analysis, and thus marker genotypes could alternatively have been imputed to the largest SNP array. How the inclusion of a residual polygenic effect in a model interacts with genotype imputation when different SNP arrays are used, would be an interesting topic for future studies.

In this paper, only phenotypes and genotypes on the sire line and the three-way crossbred pigs were considered. A more general approach would be to incorporate genotypes and phenotypes on the two maternal lines (in this paper Danbred Yorkshire and Danbred Landrace) and consider these as additional correlated traits, i.e. instead of a bivariate model as used here, a four-variate model would be used. Such a model would be an extension of the model in Christensen et al. [26] and Sevillano et al. [15] by including dominance and inbreeding depression genetic effects, and would contain a large number of parameters. It should be noted that in Sevillano et al. [15], estimation of parameters was not feasible in the full model for computational reasons, and had to be done in two-trait submodels and then combined in an ad hoc manner. Thus an extension of that model by including dominance effects would likely be very computationally demanding in practice.

## Conclusions

In this paper, we present a model for genomic evaluation in a terminal three-way crossbreeding system when information on the purebred sire line and three-way crossbred pigs is available. This model is a modification of previously reported models, accounts for both additive and dominance genotypic effects, and provides flexibility through genetic correlations for these effects between purebreds and crossbreds. Equations were derived for additive genetic parameters as functions of parameters in the model, allele frequencies, and genotype frequencies, for EBV for purebred and crossbred performances as functions of estimated effects in the model, allele frequencies and genotype frequencies, and for accuracies of EBV as functions of allele frequencies, and genotype frequencies and submatrices of the inverse coefficient matrix in the mixed model equations. The methods presented in this paper will be useful for practical implementation of genomic selection for both purebred and crossbred performances in a three-way crossbreeding system.

## Additional file

**Additional file 1.** Investigation of predictive performance.

## Data Availability

The data used in this study are not publicly available.

## Appendix

Here, we describe how to compute the prediction error variance (PEV) of EBV. For simplicity, the description is focused on PEV of EBV for purebred performance, and the differences for PEV of EBV for crossbred performance are mentioned at the end of the section.

The vector of breeding values for purebred performances is defined by ${\mathbf {BV}}_p=\mathbf {Z}_{p}(\mathbf {a}_{p}+(\mathbf {d}_{p}-(\eta _{p}/k)\mathbf {1})*(\mathbf {q}_{p}-\mathbf {p}_{p}))$. The prediction error variance–covariance matrix is ${\text {Var}}({\mathbf {BV}}_p-{\mathbf {EBV}}_p)$, which can be obtained as a function of the prediction error variance–covariance matrix of SNP effects and inbreeding depression effects as follows. Let $\mathbf {C}$ be the inverse of the coefficient matrix in the mixed model equations for the SNP effects model, and extract columns and rows of this matrix corresponding to inbreeding depression effects, additive SNP effects and dominance SNP effects for purebred performance. Then,

$$\begin{aligned} {\text {Var}}\left[ \begin{array}{c} \eta _{p} - {\hat{\eta }}_{p} \\ \mathbf {a}_{p} - {\hat{\mathbf {a}}}_{p} \\ \mathbf {d}_{p} - {\hat{\mathbf {d}}}_{p} \end{array} \right] = \left[ \begin{array}{ccc} i_0 &{} \mathbf {i}_a &{} \mathbf {i}_d \\ \mathbf {i}^{{\mathrm{T}}}_a &{} \mathbf {B}_{aa} &{} \mathbf {B}_{ad} \\ \mathbf {i}^{{\mathrm{T}}}_d &{} \mathbf {B}_{ad}^{{\mathrm{T}}} &{} \mathbf {B}_{dd} \end{array} \right] , \end{aligned}$$

where the submatrices correspond to columns and rows of matrix $\mathbf {C}$ for inbreeding depression effects, additive SNP effects and dominance SNP effects for purebred performances, respectively, and superscript T refers to the transpose of a matrix. From the equation:

$$\begin{aligned} {\mathbf {BV}}_p-{\mathbf {EBV}}_p =\mathbf {Z}_{p}(\mathbf {a}_{p}-{\widehat{\mathbf {a}}}_{p}+(\mathbf {d}_{p}-{\widehat{\mathbf {d}}}_{p}-(\eta _{p}-{\hat{\eta }}_{p})\mathbf {1}/k)*(\mathbf {q}_{p}-\mathbf {p}_{p})), \end{aligned}$$

we obtain

$$\begin{aligned} {\text {Var}}({\mathbf {BV}}_p-{\mathbf {EBV}}_p)&=\mathbf {Z}_{p}\big (\mathbf {B}_{aa}+(\mathbf {B}_{ad}-\mathbf {i}_a\mathbf {1}^{{\mathrm{T}}}/k){\varvec{\Delta }}+{\varvec{\Delta }}(\mathbf {B}_{ad}^{{\mathrm{T}}}-\mathbf {1}\mathbf {i}_a^{{\mathrm{T}}}/k) \\&\quad +{\varvec{\Delta }}(\mathbf {B}_{dd}-\mathbf {i}_d\mathbf {1}^{{\mathrm{T}}}/k-\mathbf {1}\mathbf {i}_d^{{\mathrm{T}}}/k+i_0\mathbf {1}\mathbf {1}^{{\mathrm{T}}}/k^2){\varvec{\Delta }}\big )\mathbf {Z}_{p}^{{\mathrm{T}}}, \end{aligned}$$

where ${\varvec{\Delta }}$ is the diagonal matrix with diagonal elements equal to elements in $\mathbf {q}_{p}-\mathbf {p}_{p}$.

For crossbred performance, the prediction error variance–covariance matrix is similar, except that the submatrices of matrix $\mathbf {C}$ correspond to inbreeding depression effects, additive genotypic effects and dominance genotypic effects for three-way crossbreds instead of purebred sire line, and the diagonal elements of matrix ${\varvec{\Delta }}$ are equal to the elements of $\mathbf {r}_{cs}$ as defined in Eq. (7).
