# Correction to: A bivariate genomic model with additive, dominance and inbreeding depression effects for sire line and three-way crossbred pigs

**DOI:** 10.1186/s12711-020-00541-x

**Published:** 2020-05-06

**Authors:** Ole F. Christensen, Bjarne Nielsen, Guosheng Su, Tao Xiang, Per Madsen, Tage Ostersen, Ingela Velander, Anders B. Strathe

**Affiliations:** 1 Center for Quantitative Genetics and Genomics, Aarhus University, Blichers Allé 20, 8830 Tjele, Denmark; 2 SEGES, Danish Pig Research Centre, Danish Agriculture & Food Council F.m.b.A., Axelborg, Axeltorv 3, 1609 Copenhagen V, Denmark; 3 College of Animal Sciences and Technology, Huazhong Agricultural University, No. 1 Shizihan St., Hongshan District, Wuhan, 430070 Hubei People’s Republic of China; 4 Quantitative Clinical Pharmacology, Novo Nordisk A/S, Vandtårnsvej 108, 2860 Søborg, Denmark

## Correction to: Genet Sel Evol (2019) 51:45 10.1186/s12711-019-0486-2

**Background**

In Christensen et al. [1], an error was made. In the paper, we claim that genetic variance and breeding values for crossbred performance of sire line boars should be computed from allele substitution effects for sire line boars when mated to a crossbred sow population, and that these should contain a correction term to account for the fact that the population of crossbred sows is not in Hardy–Weinberg equilibrium. This is not correct.

As explained by Duenk [2] (see in particular sub-Sect. 4.2.1), when we are interested in the value that is transmitted to crossbred offspring, the appropriate definition to use is the average value of transmitted alleles to the offspring. In the context of the three-way crossbreeding considered in Christensen et al. [1], sire line alleles transmitted to crossbreds will always pair with an allele from the sow population. Thus, the average effect for crossbred performance is $\alpha + \left( {p_{s} - q_{s} } \right)d$, where $\alpha$ is the additive effect, $d$ is the dominance effect, and $p_{s}$ and $q_{s} = 1 - p_{s}$ are allele frequencies of the sow population. The implication is that there should not be a correction term to account for the population of crossbred sows not being in Hardy–Weinberg equilibrium. Thanks to Pascal Duenk from Wageningen University for pointing this out.

The aim of this correction paper is to present the correct formulas for the genetic variance and covariance, and breeding values in “Methods” section, and to present updated results for the data analysis in the Results section. The updated results are very similar to the previous ones, with only changes in final decimals, and hence they do not cause any changes in the Discussion or Conclusions sections. Below, the equation, table and figure numbers are consistent with those in Christensen et al. [1], and the written text is also consistent to the extent that it makes sense, except that we have followed Duenk [2], and now we use the term “average effect” instead of “allele substitution effect”.

We apologize for any inconvenience.

## Methods

### Additive genetic variance and covariance

The additive genetic variance for crossbred performance (mating sire line with sows from another population) is obtained from the vector of average effects for sire line boar alleles when mated to the specific population. The vector of average effects is expressed as:

6 $${\varvec{\upalpha}}_{c} = {\mathbf{a}}_{c} + (\left( { - \eta_{c} /k)1 + {\mathbf{d}}_{c} } \right) \times {\mathbf{r}}_{cs} ,$$

with

7 $${\mathbf{r}}_{cs} = ({\mathbf{q}}_{cs} - {\mathbf{p}}_{cs} ),$$

where ${\mathbf{p}}_{cs}$ and ${\mathbf{q}}_{cs}$ are vectors of frequencies of the first and second allele in crossbred sows, respectively. The resulting expression for the additive genetic variance for crossbred performance of sire line boars (mating with crossbred sows) is:

8 $$\sigma_{g,c}^{2} = \mathop \sum \limits_{j} 2p_{p}^{j} q_{p}^{j} \sigma_{a,c}^{2} + \mathop \sum \limits_{j} 2p_{p}^{j} q_{p}^{j} \left( {r_{cs}^{j} } \right)^{2} \left( {\sigma_{d,c}^{2} + \eta_{c}^{2} /k^{2} } \right),$$

where $r_{cs}^{j}$ is the $j{\text{th}}$ element of the vector defined in Eq. (7).

The additive genetic covariance between purebred and crossbred performances is obtained from covariances between average effects, which becomes:

9 $$\begin{aligned} \sigma_{g,pc} & = \mathop \sum \limits_{j} 2p_{p}^{j} q_{p}^{j} {\text{E}}\left[ {\alpha_{p}^{j} \alpha_{c}^{j} } \right] \\ & = \mathop \sum \limits_{j} 2p_{p}^{j} q_{p}^{j} \sigma_{a,pc} + \mathop \sum \limits_{j} 2p_{p}^{j} q_{p}^{j} \left( {q_{p}^{j} - p_{p}^{j} } \right)r_{cs}^{j} \left( {\sigma_{d,pc} + \eta_{p} \eta_{c} /k^{2} } \right) \\ \end{aligned}$$

### Breeding values for crossbred performance

Breeding values of sire line boars for three-way crossbred performance are obtained from the average effects of sire line boar alleles for crossbred performance as shown in Eq. (6), and can therefore be expressed as ${\mathbf{BV}}_{c} = {\mathbf{Z}}_{p} \left( {{\mathbf{a}}_{c} + \left( {\left( { - \eta_{c} /k} \right)1 + {\mathbf{d}}_{c} } \right) \times {\mathbf{r}}_{cs} } \right)$, where ${\mathbf{r}}_{cs}$ is defined in Eq. (7). Thus, from the SNP effects model in Christensen et al. [1], EBV for three-way crossbred performance of sire line boars are equal to:

13 $${\mathbf{EBV}}_{c} = {\mathbf{Z}}_{p} \left( {{\hat{\mathbf{a}}}_{c} + \left( {\left( { - \hat{\eta }_{c} /k} \right)1 + {\hat{\mathbf{d}}}_{c} } \right) \times {\mathbf{r}}_{cs} } \right),$$

and accuracies can be obtained from prediction error variance (PEV) as:

$$acc_{c,i} = 1 - \sqrt {PEV_{c,i} /Var\left( {BV_{c,i} } \right)} ,$$

where $\left( {BV_{c,i} } \right) = \left( {{\mathbf{Z}}_{p} {\mathbf{Z}}_{p}^{T} } \right)_{ii} \sigma_{a,c}^{2} + \left( {{\mathbf{Z}}_{\varvec{p}} {\varvec{\Delta}}_{c}^{2} {\mathbf{Z}}_{p}^{T} } \right)_{ii} \left( {\sigma_{d,c}^{2} + \eta_{c}^{2} /k^{2} } \right)$, with ${\varvec{\Delta}}_{c}$ being a diagonal matrix with elements ${\mathbf{r}}_{cs}$, and with $PEV_{c}$ computed as explained in the Appendix in Christensen et al. [1], but using ${\mathbf{r}}_{cs}$ as defined in Eq. (7).

## Results

Estimates of additive genetic variances, covariance and correlation (with associated standard errors) for purebred and crossbred performances for each trait are in the table below, equivalent to Table 3 in Chritensen et al. [1]. Estimates of additive genetic correlations between purebred and crossbred performances ranged from 0.76 for ADG to 0.97 for BF.

**Table 3:** Additive genetic parameters and heritabilities. Additive genetic parameters for the four traits are computed from model parameter estimates in Table 2 in Christensen et al. [1]. Concerning parameters $\sigma_{g,p}^{2}$ and $h_{p}^{2}$, we refer to Table 3 in Christensen et al. [1]. Variance for crossbred performance: $\sigma_{g,c}^{2}$ computed using Eq. (8), covariance between purebred and crossbred performances: $\sigma_{g,pc}$ computed using Eq. (9), correlation between purebred and crossbred performances: $\rho_{g,pc}$, and heritability $h_{c}^{2} = \sigma_{g,c}^{2} /(\sigma_{a,c}^{2} \overline{\delta G}_{c} \sigma_{d,c}^{2} + \overline{\delta D}_{c} \sigma_{l,c}^{2} + \sigma_{e,c}^{2} ),$where $\overline{\delta G}_{c}$ and $\overline{\delta D}_{c}$ are averages of diagonal elements in matrices ${\mathbf{G}}_{c}$ and ${\mathbf{D}}_{c}$ in Christensen et al. [1], respectively. BF: ultra-sound recorded backfat thickness, CONF: overall conformation score, ADG: average daily gain, FCR: feed conversion ratio

	BF	CONF	ADG	FCR
$$\sigma_{g,p}^{2}$$				
$\sigma_{g,c}^{2}$	0.129 (0.016)	0.038 (0.009)	0.0026 (0.0004)	0.0026 (0.0006)
$\sigma_{g,pc}$	0.102 (0.010)	0.042 (0.009)	0.0013 (0.0003)	0.0022 (0.0005)
$\rho_{g,pc}$	0.97 (0.07)	0.83 (0.16)	0.76 (0.17)	0.88 (0.17)
$$h_{p}^{2}$$				
$h_{c}^{2}$	0.21 (0.02)	0.09 (0.02)	0.15 (0.02)	0.10 (0.02)

EBV of Duroc boars for purebred and crossbred performances are in Fig. 1, and accuracies of EBV for purebred and crossbred performances of Duroc boars are in Fig. 2.

**Fig. 1:**
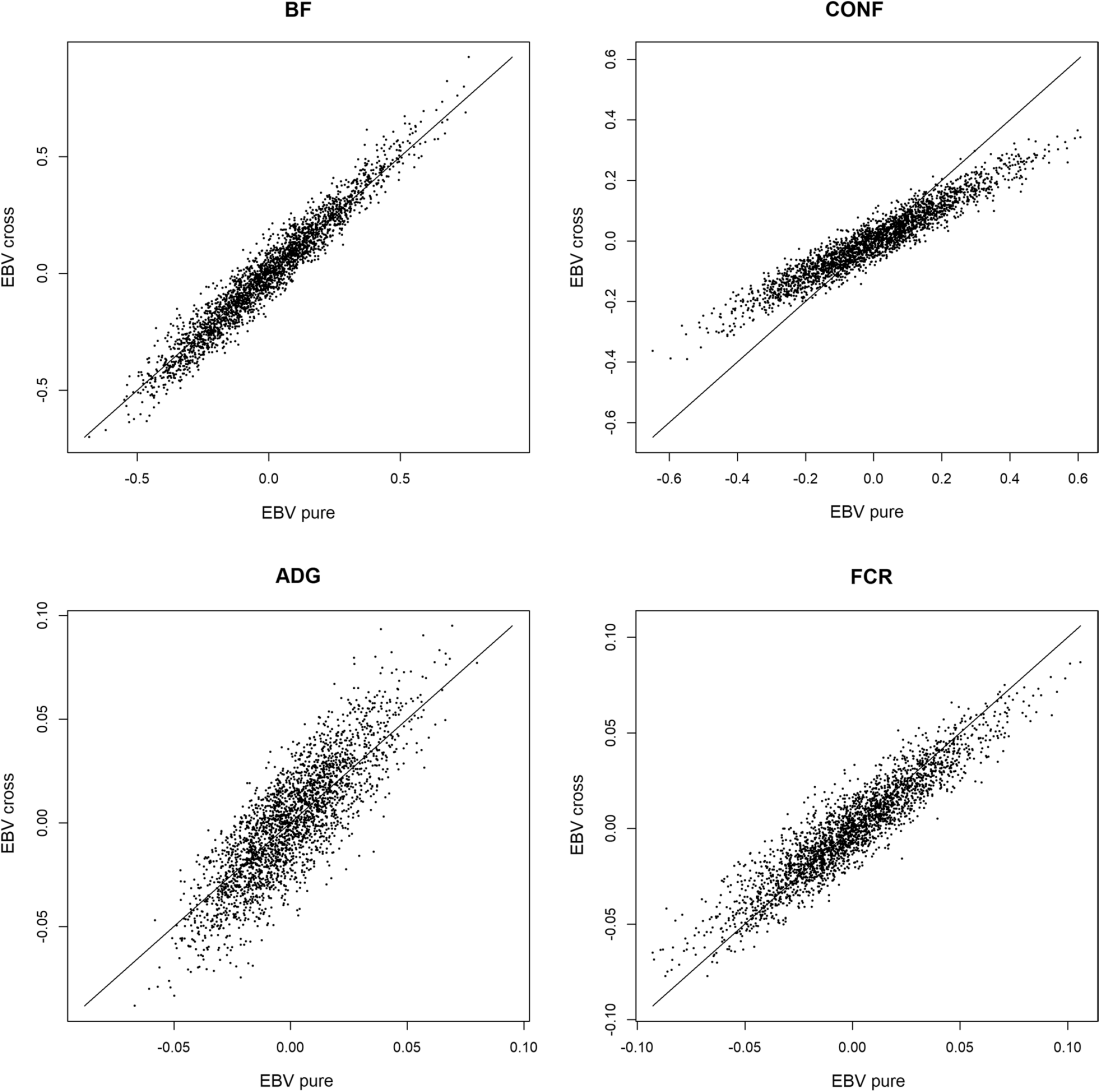
EBV for purebred and crossbred performances in Duroc boars. *BF* ultra-sound recorded backfat thickness, *CONF* overall conformation score, *ADG* average daily gain, *FCR* feed conversion ratio

**Fig. 2:**
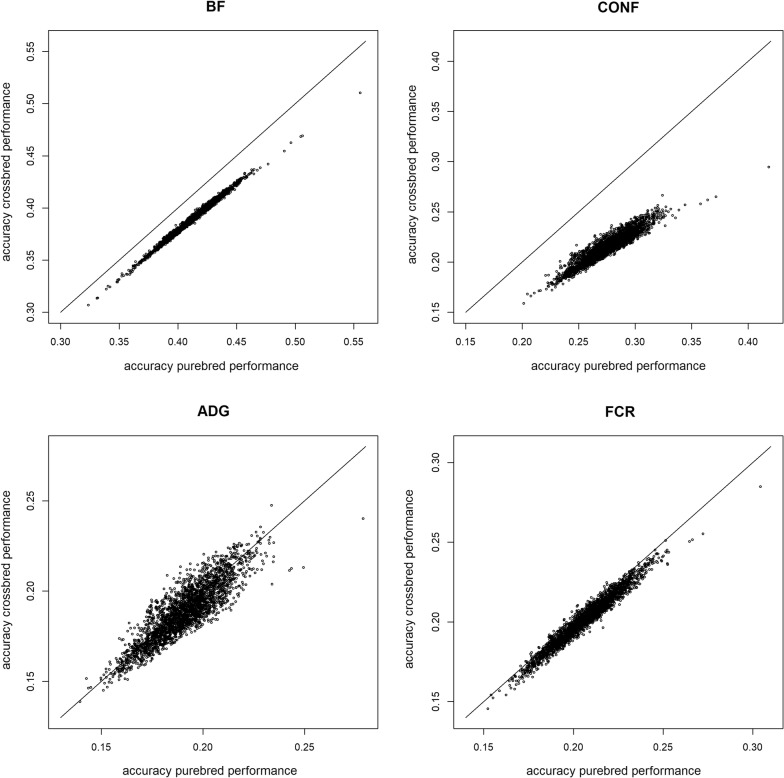
Accuracies of EBV for purebred and crossbred performances in Duroc boars. *BF* ultra-sound recorded backfat thickness, *CONF* overall conformation score, *ADG* average daily gain, *FCR* feed conversion ratio
