# Supplementary material for: A bivariate genomic model with additive, dominance and inbreeding depression effects for sire line and three-way crossbred pigs
Source: Genet Sel Evol. 2019 Aug 19;51:45. doi: 10.1186/s12711-019-0486-2 (PMC6701075; doi:10.1186/s12711-019-0486-2)
Supplement: Supplementary file 1 — Additional file 1. Investigation of predictive performance. [file 12711_2019_486_MOESM1_ESM.pdf]

Additional file for the *Genetics Selection Evolution* paper “A bivariate genomic model with additive, dominance and inbreeding depression effects for sire line and three-way crossbred pigs” by Ole F. Christensen, Bjarne Nielsen, Guosheng Su, Tao Xiang, Per Madsen, Tage Ostersen, Ingela Velander and Anders B. Strathe

Investigation of predictive performance

The following four models were compared

ModelADI: The model in formula (1) in the paper, i.e. model with additive, dominance and inbreeding depression effects for sire line and for crossbreds.

ModelAD: As ModelADI, but without inbreeding depression effects, i.e. no regression on inbreeding coefficient for sire line part and for crossbred part of the model.

ModelAI: As ModelADI, but without dominance genetic effects, i.e. no genotypic dominance effect for sire line part and for crossbred part of the model

ModelA: As ModelAI, but without dominance genetic effects, i.e. no genotypic dominance effect and no inbreeding depression effect for sire line part and for crossbred part of the model

Training data set consisted of records on animals that entered the test before 1st April 2015, and validation data consisted of records on animals that entered at this date or after.

Predictive performance for crossbred data was investigated by correlation $cor(y_{cc}, g_c)$, where $g_c = f_c\eta_c + u_c + v_c$ are the total genetic effect on crossbreds in validation data set estimated using the training data set, and y_{cc} are the corrected phenotypic records (removing non-genetic fixed effects and the random litter effect) of crossbreds in the validation data set estimated using the full data set and model in (1). Predictive performance for purebred performance was investigated similarly.

Whether differences were statistically significant was evaluated using Hotelling-Williams t-test (Dunn and Clarke, 1971) at level 5%

Results for prediction of crossbred performance are as follows

	BF	CONF	ADG	FCR
modelADI	0.406	0.190	0.272	0.179
modelAD	0.406	0.190	0.270	0.183
modelAI	0.404	0.190	0.271	0.180
modelA	0.404	0.190	0.270	0.185

None of these differences are statistically significant.

Results for prediction of purebred performance are as follows

	BF	CONF	ADG	FCR

modelADI	0.345	0.268	0.161	0.159 $_{a,b}$
modelAD	0.342	0.269	0.164	0.165 $_{a}$
modelAI	0.345	0.268	0.143	0.129 $_{c}$
modelA	0.342	0.269	0.144	0.138 $_{b,c}$

where subscripts a,b,c for FCR indicate which differences are statistically significant. For BF, CONF and ADG none of these differences are statistically significant.

Dunn, O.J. and Clarke, V. (1971). Comparison of tests of the equality of dependent correlation coefficients. *J. Amer. Stat. Assoc.* **66**:904-908.
